# Sleep and thermoregulation

**DOI:** 10.1016/j.cophys.2019.11.008

**Published:** 2020-06

**Authors:** Edward C Harding, Nicholas P Franks, William Wisden

**Affiliations:** 1 Department of Life Sciences, Imperial College London, South Kensington, SW7 2AZ, UK; 2 Centre for Neurotechnology, Imperial College London, SW7 2AZ, UK; 3 UK Dementia Research Institute at Imperial College London, UK

## Abstract

In homeothermic animals sleep preparatory behaviours often promote thermal efficiency, including warmth-seeking, adopting particular postures (curling up, head tucking) and nest building, all promoting warmer skin microclimates. Skin warmth induces NREM sleep and body cooling via circuitry that connects skin sensation to the preoptic hypothalamus. Coupling sleep induction and lower body temperature could serve to minimise energy expenditure or allow energy reallocation. Cooling during NREM sleep may also induce transcriptional changes in genes whose products facilitate housekeeping functions or measure the time spent sleeping.

**Current Opinion in Physiology** 2020, **15**:7–13

This review comes from a themed issue on **Physiology of sleep**

Edited by **Vladyslav Vyazovskiy** and **Jenny Morton**

For a complete overview see the Issue and the Editorial

Available online 26th November 2019

**https://doi.org/10.1016/j.cophys.2019.11.008**

2468-8673/© 2019 The Authors. Published by Elsevier Ltd. This is an open access article under the CC BY license (http://creativecommons.org/licenses/by/4.0/).

## Introduction

Sleep takes place whether animals are warm or cold blooded [1]. Mammals and birds are homeotherms. They generate heat through their metabolism and maintain body temperature above that of the ambient surroundings [2]. But when homeotherms enter NREM sleep, they cool down. Thirty years ago, McGinty and Szymusiak explored this correlation and suggested that cooling served important functions, rather than simply being the less interesting consequence of not moving [3]. They speculated that these functions included ‘*energy conservation, restoration of fatigable cerebral processes, avoidance of biophysical disorders resulting from sustained high temperature, and the immune response*’ [3]. But even now, there are no definitive answers concerning the role of temperature in sleep function. In this review, we consider the recent advances in understanding the relationship between thermoregulation and sleep.

## Thermoregulation over the sleep wake cycle

Body temperature is under circadian control [4]. Even human patients confined to ‘bed-rest’, where the effects of physical activity are minimised, maintain stable 24-hour temperature cycles of approximately 1°C [5]. Two hours before falling asleep, our core temperature starts to decrease under circadian control [6]. The likelihood of the first bout of NREM sleep is highest when the rate of body temperature decline is maximal [6]. But circadian changes in body temperature can be uncoupled from changes brought about directly by sleep entry [4]. In experiments where the circadian rhythm is desynchronised from the sleep cycle, the effect of sleep itself on body temperature becomes clear: core temperature drops on every transition to NREM sleep [4].

In mice, core body temperature decline also coincides with the point at which they are most likely to sleep, just as is seen in humans [6]. Thus, it is not just circadian phase and physical activity that determine core and brain temperature, but instead, the primary drivers are sleep-wake states themselves [7]. More recently, Hoekstra *et al.* also found that sleep state was a larger determinant of brain cortical temperature than locomotion [8^••^]. On each transition from wake to NREM, cortical temperature decreases by about 0.2°C, but rises again quickly in the next wakefulness episode [8^••^]. On the other hand, REM sleep is accompanied by an increase in brain temperature of approximately 0.1–0.2°C, although this is smaller than that seen in wake [8^••^,9^••^].

In humans, increases in circulating melatonin correlate with sleep onset, subjective sleepiness and a decline in core temperature [10]. In CBA mice melatonin is highest in the late dark phase, that is, the late waking portion of the day [11]. Most laboratory strains of mice, however, cannot synthesise melatonin [11], and so melatonin can play no essential role in regulating temperature changes and the time of sleep onset in mice. Rats become hypothermic when injected with melatonin in the light phase, but not in the dark phase [12], suggesting a complex and species-dependent relationship between circulating melatonin, sleep induction and temperature decline.

## Nesting: microclimates for energy conservation

Recently, research on sleep preparatory behaviours, for example, nest building in the case of mice, suggests that such behaviours require dedicated neuronal circuitry. This engages before sleep onset, requiring inhibition of ventral tegmental area dopamine neurons [13,14]. Nesting allows a sleeping environment close to thermoneutrality, where core temperature can be maintained with minimum energy expenditure. Given a temperature preference, mice will also choose nesting sites in warmer environments, closer to thermoneutrality [15^••^], where they will spend 85% of the light period [16^•^,^17^]. However, when nest site temperatures rise above thermoneutrality, the nesting material becomes unnecessary and nest quality deteriorates [18^•^]. The importance of nesting insulation for smaller mammals cannot be overstated. Mice living at 10°C expend three and a half times more energy than those close to thermoneutrality and consume three times more food to compensate [19]. The presence of nesting material reduces this food consumption and can even reduce litter mortality [20] (reviewed in Ref. [6]).

Sleep posture is also important in energy conservation. For example, to recover from long migration bouts, garden warblers adopt energy saving postures during sleep, by tucking their head into their body, despite increased risk of predation [21^•^]. This has parallels to the sleeping postures and curling up behaviour common to mammals. Given the prevalence of conserved behaviours to save energy during sleep, we suggest a neuronal mechanism may exist that promotes sleep optimised towards conserving energy at thermoneutral temperatures.

## Getting ready to sleep: microclimates and the warm bath effect

In addition to energy conservation, there is another reason why the warmth provided by nesting and adopting specific sleep postures, that is, curling up, or for humans, changing into night clothes and getting under the duvet, could be important. This insight comes from the ‘warm bath effect’. Warming before sleep, usually from a warm bath or shower, promotes shorter sleep latencies, longer initial sleep episodes and even ‘deeper’ sleep as measured by EEG [22^•^,^23^]. Similarly, specific warming of the hands and feet promotes NREM sleep induction [24, 25, 26, 27] (reviewed in Ref. [28]). The ‘warm bath effect’ is a clear phenomenon: a meta-analysis of 13 human trials concluded that water-based passive warming for as little as ten minutes, between one and two hours before sleep, shortens sleep latency by approximately 36% [22^•^]. This is mechanistically consistent with an increase in peripheral vasodilation observed in several human trials, that results in a decrease in core temperature and a corresponding decrease in sleep latency [22^•^,^29^, ^30^, ^31^].

**Figure 1:**
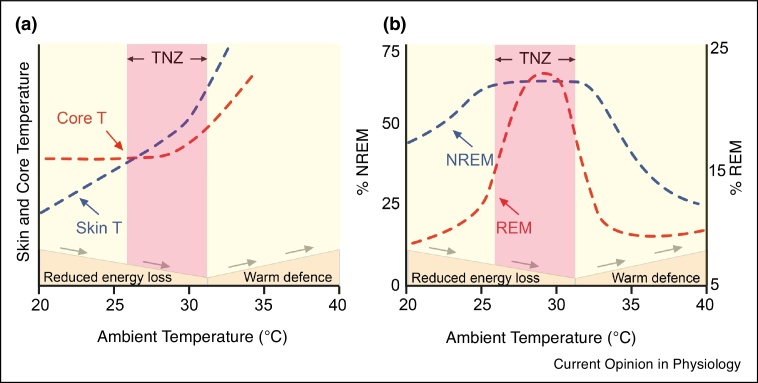
The relationship between sleep and ambient temperature. **a**, The changing relationship of skin to core temperature as mice approach the thermoneutral zone (TNZ). This has similarities to the distal-proximal gradient in humans where vasodilation in distal regions increases peripheral skin temperature and facilitates heat redistribution from the core. Having reached the cooler night-time temperature, the formation and maintenance of warm microclimates minimises energy loss while maintaining vasodilation. **b**, The structure of sleep is sensitive to ambient temperature. In mice, increasing ambient temperature promotes NREM sleep until the upper threshold of the thermoneutral zone where it declines sharply most likely due to heat stress. REM sleep is maximised in a narrow thermal window that appears to align with the TNZ. It should be noted that the thermoneutral pulsing method employed in [9] did not fully replicate warm-induced increases in NREM sleep. Adapted from [9,18,^28^,^53^,57,^62^].

Nesting or sleeping under blankets could be a deliberate thermoregulatory behaviour that promotes local skin warming, or a microclimate of skin warmth, permissive for sleep and it is this process that the ‘warm bath effect’ mimics [15^••^]. Seen in another way, in preparation for sleep, mammals minimise the gradient between the skin and core temperature to reduce the energy lost as heat to the environment [18^•^] (Figure 1a). In clinical studies, this is approximated by the distal-to-proximal gradient that increases (towards zero) as sleep approaches [32]: that is, during wake, the (proximal) torso is warmer than the (distal) hands or feet, but before sleep the hands and feet become progressively warmer until they equal the torso. By using a duvet and/or night clothes, people form skin microclimates of around of 33 to 35°C — between 2 to 3°C warmer than during waking — and core temperature also falls by 1°C resulting in a thermal gradient change of as much as 4°C before sleep [29,32,33]. This could explain why increasing the ambient temperature toward the thermal neutral point enhances NREM sleep in rodents [15^••^,^34^] (Figure 1b). In support of a microclimate mechanism, capsaicin ablation of skin and brain thermoreceptors in rats eliminates warmth induced increases in sleep [34]. Cation channels activated by warming are present on sensory afferents in the skin but also on many neurons in the brain. Although direct hypothalamic warming promotes NREM sleep [35, 36, 37, 38], mild ambient warming does not produce clear changes in brain temperature [3] (reviewed in Ref. [39]), suggesting that NREM sleep induction by ambient warmth relies on the ion channels in the skin sensory afferents.

Further support for local skin warming promoting NREM sleep comes from uncoupling protein 1 (UCP-1) KO mice [40^•^]. The skin can be warmed not just by ambient temperature but also by brown adipose tissue (BAT) thermogenesis. UCP1 is expressed in BAT and is required for the heat production capacity of brown adipocyte mitochondria. Pharmacological stimulation of BAT thermogenesis with β3-adrenergic agonists (the β3 adrenergic receptor is expressed on BAT) enhances NREM sleep [41^•^]. In normal mice, administering inflammation-promoting agents (TNFα, IL-1β, lipopolysaccharide and clodronate-containing liposomes) induces a biphasic response: 6–12 hours of body cooling and extra NREM sleep, followed by 12 hours of hyperthermia (fever) and normal amounts of NREM sleep. However, in UCP-1KO mice, the fever-promoting agents no longer induce the extra NREM sleep or initial hypothermia [40^•^]. One interpretation is that these agents induce NREM sleep, and perhaps the associated hypothermia, via local skin warming from BAT stimulation.

Adult humans have cold-inducible depots of BAT, although the metabolic significance of human non-shivering thermogenesis remains contentious [42]. In neonates, the significance is clear and BAT thermogenesis provides compensation for increased surface area-to-volume ratio and insufficient skeletal muscle mass [43]. Hence, BAT may have a more important role in neonatal sleep that more closely mirrors BAT contributions to sleep in rodents.

A final point to note is that, in rodents, certain types of acute stress (e.g. social defeat stress, fighting and restraint) promotes NREM sleep [44,45]. However, acute stress in mice also induces BAT thermogenesis [46] (sleep in the cited study was not investigated). This stress-induced BAT thermogenesis could feasibly promote sleep, possibly explaining the link between acute stress and increased sleep.

## Circuitry of sleep and temperature regulation

**Figure 2:**
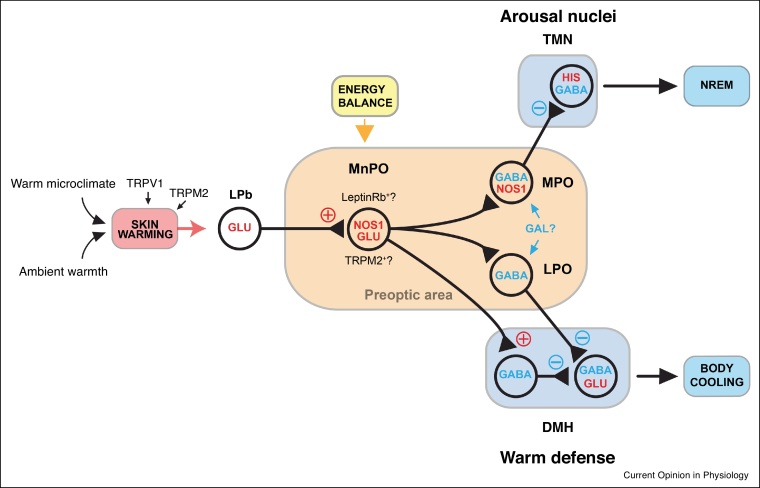
Possible circuit arrangements for the detection and integration of warm thermal information into sleep-promoting circuitry. Warmth is detected by TRPM2 channels on neuronal afferents in the skin and this information is transmitted to the lateral parabrachial nucleus (LPb) and on to nitrergic-glutamate neurons in the MPO/MnPO hypothalamus. Both nitrergic and glutamatergic populations have a degree of heterogeneity and express a mixture of transient receptor potential melastatin 2 (TRPM2) channels and leptin receptors. These nitrergic-glutamate neurons in MPO/MnPO can initiate warm defence, probably through innervation of dorsal medial hypothalamus (DMH) neurons, but they also promote sleep. This circuit could involve short range innervation of local GABAergic populations or longer projections to LPO GABA/galanin neurons or directly to arousal or sleep promoting regions. Adapted from [6,15^••^,^47^,^48^,^50^,57^••^,58^••^].

Sensory neurons in the skin use transient receptor potential channels (TRP) to detect increases in ambient temperature [47,48]. This information reaches the lateral parabrachial nucleus (LPb) in the brainstem and is transmitted to the MnPO and MPO nuclei [39,49, 50, 51, 52]. The glutamatergic neurons in the MnPO and MPO then signal to downstream targets including the dorsal medial hypothalamus and rostral raphe pallidus to induce, depending on species, vasodilation, sweating, panting and the down regulation of BAT [39,49, 50, 51, 52] (Figure 2). Specific hypothalamic cell types in MPO, such as BDNF-, PACAP- and TRPM2-expressing neurons, can be activated by external warming and when optogenetically or chemogenetically activated, result in hypothermia [39,49, 50, 51] but none of these studies looked at what happened to the vigilance state (e.g. sleep). Furthermore, the external temperatures (e.g. 38°C) used to activate these hypothermia-inducing neurons were considerably above thermoneutrality and are likely too hot to promote sleep in rodents [53]. So, it is unclear at the moment if the BDNF-neurons and PACAP-neurons are involved in sleep induction by ambient warmth.

Recently some of the mechanisms by which warmth-induced sleep may take place have been discovered. Placing mice in a warm environment at their thermoneutral temperature activates hypothalamic median and medial preoptic (MnPO/MPO) hypothalamic glutamate/NOS1 neurons [15^••^], presumably by the route described in the above paragraph. These glutamate/NOS1 neurons can induce simultaneous hypothermia and NREM sleep (Figure 2) [15^••^]. To induce hypothermia, the glutamate/NOS1 neurons would innervate the previously described circuitry of warm defence. To induce NREM sleep, they may innervate sleep-promoting GABA neurons, also located in MPO [15^••^]. When these GABA neurons are activated, there is little hypothermia induced, demonstrating that NREM sleep can be artificially dissociated from hypothermia. The sleep-promoting GABA neurons could also be nitrergic, as RNA profiling indicates that GABAergic NOS1 neurons are also present in MPO/MnPO [15^••^,54^••^] (Figure 2).

The MnPO sends both inhibitory and excitatory connections to the LPO region. Thus, skin warmth-activated MnPO/MPO glutamate/NOS1 neurons could potentially innervate both galanin/GABA neurons in the LPO hypothalamus, which have been long postulated to induce NREM sleep [55,56], as well as GABA neurons in MPO (see Figure 2). When chemogenetically or optogenetically activated, galanin neurons in LPO do, indeed, induce NREM sleep [57^••^,58^••^], but also concomitantly hypothermia [57^••^,58^••^,^59^]. When galanin neurons in LPO are lesioned, mice have slightly increased amounts of sleep, but sleep becomes highly fragmented [58^••^], and the mice have difficulty catching up on lost sleep after sleep deprivation, that is, their sleep homeostasis becomes impaired [58^••^]. Consistent with the chemogenetic stimulation results [57^••^], mice with lesioned galanin neurons develop chronic hyperthermia, retaining their usual diurnal variation in body temperature, but with the temperature shifted up by several degrees [58^••^]. Overall, these combined effects are unlikely to result from a single type of LPO galanin or MPO GABAergic neuron. Molecular phenotyping of the PO area has revealed multiple types of galanin neuron which are intermingled; most galanin neurons are GABAergic, but some co-release GABA and glutamate, and one population utilises GABA and monoamines [54^••^]. The reason that mice with genetic lesioning of LPO galanin neurons actually sleep more in baseline conditions is not clear, but given sleep’s importance, it could be a compensatory mechanism by other elements of the sleep-promoting circuitry [58^••^].

## Thermal theories of REM sleep

During REM sleep in rodents, body temperature is not regulated. In contrast to NREM sleep, REM sleep is characterised by brain temperature rises resulting from the relative increase in warmer vertebral, over carotid artery, blood flow (please see background literature for this in Ref. [8^••^]). In humans, thermoregulatory disruption during REM sleep is less clear cut: sweating responses are observed, but they are blunted in REM sleep [60]. REM sleep in rodents operates in a narrow thermal window whereby the amount of REM is maximal around thermoneutral temperatures [61,62] (Figure 1b). For instance, the percentage of REM sleep doubles as the temperature rises from 22 to 29°C, but returns to baseline at 36°C [9^••^,57^••^]. This thermal neutral preference for REM sleep is abolished in the MCH receptor 1 knockout mouse [9^••^]. Optogenetically inhibiting MCH neurons produces the same result whilst stimulation at thermoneutral temperatures further increased REM sleep [9^••^]. Hence, a mechanism that directs REM sleep initiation towards optimal thermal efficiency exists in the mouse and may support an ‘energy allocation’ hypothesis for REM sleep [9^••^,63^•^,^64^].

There is a significant relationship between disrupted REM sleep and disrupted thermoregulation in Parkinson’s disease [65]. REM sleep behaviour disorder (RSB) is a significant risk factor for Parkinson’s disease, with more than 75% of RSB patients developing Parkinson’s over a subsequent 12 year period [66]. Parkinson’s patients have lower night time core body temperatures that correlate with the severity of RSB symptoms [67]. Patients can also have disrupted sweat responses [68]. Hence, there is a need to understand the basic biology of REM sleep and temperature regulation in these patients to aid in their care.

## Why would brain cooling be an important feature of sleep?

One unanswered question is why NREM sleep and body cooling seem to be linked. Similarly, why is REM sleep different or privileged in this regard? On the scale of the whole organism this process appears optimised towards energy conservation. However, total energy savings of eight hours sleep, in a 24-hour cycle, are as small as 5–15% [64,69,70]. It is possible, instead, that sleep facilitates a reallocation of resources that cannot be achieved during wakefulness, which may amplify these energy savings by as much as 35% [63^•^,^64^]. For example, some functions of the immune system change during sleep and might be achieved more efficiently in this manner [71].

Alternatively, cooling during each NREM sleep episode may impact cellular function on a molecular level. For example, expression of Cold-Inducible RNA Binding Protein (CIRBP) and RBM3 (RNA binding motif protein 3) genes is induced at the lower temperatures encountered during bouts of NREM sleep [8^••^,^72^,^73^]. These proteins alter clock gene expression. Sleep deprivation dampens CIRBP expression and hence cooling during NREM sleep is one putative mechanism by which the time spent sleeping could be measured though altered clock gene expression [8^••^]. CIRBP ablation in mice results in reduced REM sleep and CIRBP expression could initiate sleep-specific housekeeping functions [8^••^]. RBM3 expression also has a neuroprotective role in the prion and Alzheimer’s mouse models, particularly in the hippocampus, and so may serve a similar function during sleep [74]. Longer or deeper bouts of NREM sleep, such as the recovery sleep (sleep homeostasis) following sleep deprivation, are associated with greater brain cooling of more than 2°C over one hour (Hubbard *et al.*, bioRxiv doi: 10.1101/748871). This suggests that sleep-associated cooling is homeostatic. LPO galanin neurons may play a central role in this because they both drive hypothermia and are needed for recovery sleep after sleep deprivation [58^••^]. This further empzzhasises the fundamental connection between NREM sleep and brain and body cooling.

We have described some features of sleep, such as cooling induced changes in gene expression. These could form important elements in the role of sleep; however, they may not be sufficient to describe the function of sleep. For example, in Djungarian hamsters comparisons between recovery sleep after daily torpor and recovery sleep after sleep deprivation revealed different EEG characteristics, suggesting these states are not entirely analogous [75]. Finally, we should note that these and other hypothesis of sleep function do not explain a key component of sleep — the requirement for loss of consciousness. Accounting for this characteristic will be necessary for any complete theory of sleep.

## Conflict of interest statement

Nothing declared.

## References and recommended reading

Papers of particular interest, published within the period of review, have been highlighted as:

• of special interest
•• of outstanding interest

## References

[1] Leung L.C., Wang G.X., Madelaine R., Skariah G., Kawakami K., Deisseroth K., Urban A.E., Mourrain P. (2019). Neural signatures of sleep in zebrafish. Nature.

[2] Polymeropoulos E.T., Oelkrug R., Jastroch M. (2018). Editorial: the evolution of endothermy - from patterns to mechanisms. Front Physiol.

[3] McGinty D., Szymusiak R. (1990). Keeping cool: a hypothesis about the mechanisms and functions of slow-wave sleep. Trends Neurosci.

[4] Dijk D.J., Czeisler C.A. (1995). Contribution of the circadian pacemaker and the sleep homeostat to sleep propensity, sleep structure, electroencephalographic slow waves, and sleep spindle activity in humans. J Neurosci.

[5] Mendt S., Maggioni M.A., Nordine M., Steinach M., Opatz O., Belavy D., Felsenberg D., Koch J., Shang P., Gunga H.C. (2017). Circadian rhythms in bed rest: monitoring core body temperature via heat-flux approach is superior to skin surface temperature. Chronobiol Int.

[6] Harding E.C., Franks N.P., Wisden W. (2019). The temperature dependence of sleep. Front Neurosci.

[7] Franken P., Tobler I., Borbely A.A. (1992). Sleep and waking have a major effect on the 24-hr rhythm of cortical temperature in the rat. J Biol Rhythms.

[8••] Hoekstra M.M., Emmenegger Y., Hubbard J., Franken P. (2019). Cold-inducible RNA-binding protein (CIRBP) adjusts clock-gene expression and REM-sleep recovery following sleep deprivation. eLife.

[9••] Komagata N., Latifi B., Rusterholz T., Bassetti C.L.A., Adamantidis A., Schmidt M.H. (2019). Dynamic REM sleep modulation by ambient temperature and the critical role of the melanin-concentrating hormone system. Curr Biol.

[10] Logan R.W., McClung C.A. (2019). Rhythms of life: circadian disruption and brain disorders across the lifespan. Nat Rev Neurosci.

[11] Kennaway D.J. (2019). Melatonin research in mice: a review. Chronobiol Int.

[12] Lopez-Canul M., Min S.H., Posa L., De Gregorio D., Bedini A., Spadoni G., Gobbi G., Comai S. (2019). Melatonin MT1 and MT2 receptors exhibit distinct effects in the modulation ofb ody temperature across the light/dark cycle. Int J Mol Sci.

[13] Eban-Rothschild A., Rothschild G., Giardino W.J., Jones J.R., de Lecea L. (2016). VTA dopaminergic neurons regulate ethologically relevant sleep-wake behaviors. Nat Neurosci.

[14] Eban-Rothschild A., Giardino W.J., de Lecea L. (2017). To sleep or not to sleep: neuronal and ecological insights. Curr Opin Neurobiol.

[15••] Harding E.C., Yu X., Miao A., Andrews N., Ma Y., Ye Z., Lignos L., Miracca G., Ba W., Yustos R. (2018). A neuronal hub binding sleep initiation and body cooling in response to a warm external stimulus. Curr Biol.

[16•] Gordon C.J., Puckett E.T., Repasky E.S., Johnstone A.F. (2017). A device that allows rodents to behaviorally thermoregulate when housed in vivariums. J Am Assoc Lab Anim Sci.

[17] Chan C.E., Hare M.T., Martin G.W., Gordon C.J., Swoap S.J. (2019). The heat is on: a device that reduces cold stress-induced tachycardia in laboratory mice. J Therm Biol.

[18•] Gordon C.J. (2017). The mouse thermoregulatory system: its impact on translating biomedical data to humans. Physiol Behav.

[19] Yu S., Cheng H., Francois M., Qualls-Creekmore E., Huesing C., He Y., Jiang Y., Gao H., Xu Y., Zsombok A. (2018). Preoptic leptin signaling modulates energy balance independent of body temperature regulation. eLife.

[20] Gaskill B.N., Pritchett-Corning K.R., Gordon C.J., Pajor E.A., Lucas J.R., Davis J.K., Garner J.P. (2013). Energy reallocation to breeding performance through improved nest building in laboratory mice. PLoS One.

[21•] Ferretti A., Rattenborg N.C., Ruf T., McWilliams S.R., Cardinale M., Fusani L. (2019). Sleeping unsafely tucked in to conserve energy in a nocturnal migratory songbird. Curr Biol.

[22•] Haghayegh S., Khoshnevis S., Smolensky M.H., Diller K.R., Castriotta R.J. (2019). Before-bedtime passive body heating by warm shower or bath to improve sleep: a systematic review and meta-analysis. Sleep Med Rev.

[23] Igaki M., Suzuki M., Sakamoto I., Ichiba T., Kuriyama K., Uchiyama M. (2018). Effects of bedtime periocular and posterior cervical cutaneous warming on sleep status in adult male subjects: a preliminary study. Sleep Biol Rhythms.

[24] Krauchi K., Cajochen C., Werth E., Wirz-Justice A. (1999). Warm feet promote the rapid onset of sleep. Nature.

[25] Ko Y., Lee J.-Y. (2018). Effects of feet warming using bed socks on sleep quality and thermoregulatory responses in a cool environment. J Physiol Anthropol.

[26] Oshima-Saeki C., Taniho Y., Arita H., Fujimoto E. (2017). Lower-limb warming improves sleep quality in elderly people living in nursing homes. Sleep Sci.

[27] Chiu H.Y., Lin E.Y., Chiu H.T., Chen P.Y. (2017). A feasibility randomized controlled crossover trial of home-based warm footbath to improve sleep in the chronic phase of traumatic brain injury. J Neurosci Nurs.

[28] Te Lindert B.H.W., Van Someren E.J.W. (2018). Skin temperature, sleep, and vigilance. Handb Clin Neurol.

[29] Okamoto-Mizuno K., Mizuno K., Shirakawa S. (2018). Sleep and skin temperature in preschool children and their mothers. Behav Sleep Med.

[30] Barcat L., Decima P., Bodin E., Delanaud S., Stephan-Blanchard E., Leke A., Libert J.-P., Tourneux P., Bach V. (2017). Distal skin vasodilation promotes rapid sleep onset in preterm neonates. J Sleep Res.

[31] Bach V., Delanaud S., Barcat L., Bodin E., Tourneux P., Libert J.P. (2019). Distal skin vasodilation in sleep preparedness, and its impact on thermal status in preterm neonates. Sleep Med.

[32] Kräuchi K., Cajochen C., Werth E., Wirz-Justice A. (2000). Functional link between distal vasodilation and sleep-onset latency?. Am J Physiol Regul Integr Comp Physiol..

[33] McCabe S.M., Elliott C., Langdon K., Abbiss C.R. (2018). Patterns and reliability of children’s skin temperature prior to and during sleep in the home setting. Physiol Behav.

[34] Obal F., Tobler I., Borbely A.A. (1983). Effect of ambient temperature on the 24-hour sleep-wake cycle in normal and capsaicin-treated rats. Physiol Behav.

[35] Roberts W.W., Robinson T.C.L. (1969). Relaxation and sleep induced by warming of preoptic region and anterior hypothalamus in cats. Exp Neurol.

[36] Glotzbach S.F., Heller H.C. (1976). Central nervous regulation of body temperature during sleep. Science.

[37] Szymusiak R., McGinty D. (1986). Sleep-related neuronal discharge in the basal forebrain of cats. Brain Res.

[38] McGinty D., Szymusiak R., Thomson D. (1994). Preoptic/anterior hypothalamic warming increases EEG delta frequency activity within non-rapid eye movement sleep. Brain Res.

[39] Siemens J., Kamm G.B. (2018). Cellular populations and thermosensing mechanisms of the hypothalamic thermoregulatory center. Pflugers Arch.

[40•] Szentirmai E., Kapas L. (2018). Brown adipose tissue plays a central role in systemic inflammation-induced sleep responses. PLoS One.

[41•] Szentirmai E., Kapas L. (2017). The role of the brown adipose tissue in beta3-adrenergic receptor activation-induced sleep, metabolic and feeding responses. Sci Rep.

[42] Leitner B.P., Huang S., Brychta R.J., Duckworth C.J., Baskin A.S., McGehee S., Tal I., Dieckmann W., Gupta G., Kolodny G.M. (2017). Mapping of human brown adipose tissue in lean and obese young men. Proc Natl Acad Sci U S A.

[43] Lidell M.E., Pfeifer A., Klingenspor M., Herzig S. (2019). Brown adipose tissue in human infants. Brown Adipose Tissue.

[44] Fujii S., Kaushik M.K., Zhou X., Korkutata M., Lazarus M. (2019). Acute social defeat stress increases sleep in mice. Front Neurosci.

[45] Kamphuis J., Lancel M., Koolhaas J.M., Meerlo P. (2015). Deep sleep after social stress: NREM sleep slow-wave activity is enhanced in both winners and losers of a conflict. Brain Behav Immun.

[46] Machado N.L.S., Abbott S.B.G., Resch J.M., Zhu L., Arrigoni E., Lowell B.B., Fuller P.M., Fontes M.A.P., Saper C.B. (2018). A glutamatergic hypothalamomedullary circuit mediates thermogenesis, but not heat conservation, during stress-induced hyperthermia. Curr Biol.

[47] Tan C.H., McNaughton P.A. (2018). TRPM2 and warmth sensation. Pflugers Arch.

[48] Tan C.H., McNaughton P.A. (2016). The TRPM2 ion channel is required for sensitivity to warmth. Nature.

[49] Madden C.J., Morrison S.F. (2019). Central nervous system circuits that control body temperature. Neurosci Lett.

[50] Tan C.L., Knight Z.A. (2018). Regulation of body temperature by the nervous system. Neuron.

[51] Morrison S.F., Nakamura K. (2019). Central mechanisms for thermoregulation. Annu Rev Physiol.

[52] Abbott S.B.G., Saper C.B. (2018). Role of the median preoptic nucleus in the autonomic response to heat-exposure. Temperature (Austin).

[53] Kumar D., Mallick H.N., Kumar V.M. (2009). Ambient temperature that induces maximum sleep in rats. Physiol Behav.

[54••] Moffitt J.R., Bambah-Mukku D., Eichhorn S.W., Vaughn E., Shekhar K., Perez J.D., Rubinstein N.D., Hao J., Regev A., Dulac C. (2018). Molecular, spatial, and functional single-cell profiling of the hypothalamic preoptic region. Science.

[55] Sherin J.E., Elmquist J.K., Torrealba F., Saper C.B. (1998). Innervation of histaminergic tuberomammillary neurons by GABAergic and galaninergic neurons in the ventrolateral preoptic nucleus of the rat. J Neurosci.

[56] Walter A., van der Spek L., Hardy E., Bemelmans A.P., Rouach N., Rancillac A. (2019). Structural and functional connections between the median and the ventrolateral preoptic nucleus. Brain Struct Funct.

[57••] Kroeger D., Absi G., Gagliardi C., Bandaru S.S., Madara J.C., Ferrari L.L., Arrigoni E., Munzberg H., Scammell T.E., Saper C.B. (2018). Galanin neurons in the ventrolateral preoptic area promote sleep and heat loss in mice. Nat Commun.

[58••] Ma Y., Miracca G., Yu X., Harding E.C., Miao A., Yustos R., Vyssotski A.L., Franks N.P., Wisden W. (2019). Galanin neurons unite sleep homeostasis and alpha2 adrenergic sedation. Curr Biol.

[59] Zhao Z.D., Yang W.Z., Gao C., Fu X., Zhang W., Zhou Q., Chen W., Ni X., Lin J.K., Yang J. (2017). A hypothalamic circuit that controls body temperature. Proc Natl Acad Sci U S A.

[60] Sagot J.C., Amoros C., Candas V., Libert J.P. (1987). Sweating responses and body temperatures during nocturnal sleep in humans. Am J Physiol.

[61] Cerri M., Luppi M., Tupone D., Zamboni G., Amici G. (2017). REM sleep and endothermy: potential sites and mechanism of a reciprocal interference. Front Physiol.

[62] Szymusiak R., Satinoff E. (1981). Maximal REM sleep time defines a narrower thermoneutral zone than does minimal metabolic rate. Physiol Behav.

[63•] Schmidt M.H., Swang T.W., Hamilton I.M., Best J.A. (2017). State-dependent metabolic partitioning and energy conservation: a theoretical framework for understanding the function of sleep. PLoS One.

[64] Latifi B., Adamantidis A., Bassetti C., Schmidt M.H. (2018). Sleep-wake cycling and energy conservation: role of hypocretin and the lateral hypothalamus in dynamic state-dependent resource optimization. Front Neurol.

[65] Raupach A.K., Ehgoetz Martens K.A., Memarian N., Zhong G., Matar E., Halliday G.M., Grunstein R., Lewis S.J.G. (2019). Assessing the role of nocturnal core body temperature dysregulation as a biomarker of neurodegeneration. J Sleep Res.

[66] Postuma R.B., Iranzo A., Hu M., Högl B., Boeve B.F., Manni R., Oertel W.H., Arnulf I., Ferini-Strambi L., Puligheddu M. (2019). Risk and predictors of dementia and parkinsonism in idiopathic REM sleep behaviour disorder: a multicentre study. Brain.

[67] Zhong G., Bolitho S., Grunstein R., Naismith S.L., Lewis S.J. (2013). The relationship between thermoregulation and REM sleep behaviour disorder in Parkinson’s disease. PLoS One.

[68] Swinn L., Schrag A., Viswanathan R., Bloem B.R., Lees A., Quinn N. (2003). Sweating dysfunction in Parkinson’s disease. Mov Dirsord.

[69] Jung C.M., Melanson E.L., Frydendall E.J., Perreault L., Eckel R.H., Wright K.P. (2011). Energy expenditure during sleep, sleep deprivation and sleep following sleep deprivation in adult humans. J Physiol.

[70] Hibi M., Kubota C., Mizuno T., Aritake S., Mitsui Y., Katashima M., Uchida S. (2017). Effect of shortened sleep on energy expenditure, core body temperature, and appetite: a human randomised crossover trial. Sci Rep.

[71] Imeri L., Opp M.R. (2009). How (and why) the immune system makes us sleep. Nat Rev Neurosci.

[72] Morf J., Rey G., Schneider K., Stratmann M., Fujita J., Naef F., Schibler U. (2012). Cold-inducible RNA-binding protein modulates circadian gene expression posttranscriptionally. Science.

[73] Tong G., Endersfelder S., Rosenthal L.-M., Wollersheim S., Sauer I.M., Bührer C., Berger F., Schmitt K.R.L. (2013). Effects of moderate and deep hypothermia on RNA-binding proteins RBM3 and CIRP expressions in murine hippocampal brain slices. Brain Res.

[74] Peretti D., Bastide A., Radford H., Verity N., Molloy C., Martin M.G., Moreno J.A., Steinert J.R., Smith T., Dinsdale D. (2015). RBM3 mediates structural plasticity and protective effects of cooling in neurodegeneration. Nature.

[75] Vyazovskiy V.V., Palchykova S., Achermann P., Tobler I., Deboer T. (2017). Different effects of sleep deprivation and torpor on EEG slow-wave characteristics in Djungarian hamsters. Cereb Cortex.

